# Dietary compliance in a randomized double‐blind infant feeding trial during infancy aiming at prevention of type 1 diabetes

**DOI:** 10.1002/fsn3.2389

**Published:** 2021-06-23

**Authors:** Suvi M. Virtanen, David Cuthbertson, Anita M. Nucci, Mila Hyytinen, Anne Ormisson, Marja Salonen, Tania Turrini, Elizabeth A. Cummings, Brenda Bradley, Marilyn Tanner‐Blasiar, Dorothy J. Becker, Hans K. Åkerblom, Erkki Savilahti, Jeffrey P. Krischer, Mikael Knip, the TRIGR Investigators

**Affiliations:** ^1^ Health and Well‐Being Promotion Unit Finnish Institute for Health and Welfare Helsinki Finland; ^2^ Faculty of Social Sciences/Unit of Health Sciences Tampere University Tampere Finland; ^3^ Center for Child Health Research Tampere University Hospital Tampere University Tampere Finland; ^4^ Research Development and Innovation Centre Tampere University Hospital Tampere Finland; ^5^ Pediatrics Epidemiology Center University of South Florida Tampa FL USA; ^6^ Department of Nutrition Georgia State University Atlanta GA USA; ^7^ University of Helsinki Helsinki Finland; ^8^ Department of Paediatrics University of Tartu Tartu Estonia; ^9^ The Hospital for Sick Children Toronto ON Canada; ^10^ Department of Pediatrics Dalhousie University Halifax NS Canada; ^11^ Children’s Hospital of Eastern Ontario University of Ottawa Ottawa ON Canada; ^12^ Department of Pediatrics Washington University School of Medicine St. Louis MO USA; ^13^ Children's Hospital of Pittsburgh Pittsburgh PA USA; ^14^ University of Pittsburgh Pittsburgh PA USA; ^15^ Children’s Hospital Helsinki University Hospital University of Helsinki Helsinki Finland; ^16^ Folkhälsan Research Center Helsinki Finland; ^17^ Department of Pediatrics Tampere University Hospital Tampere Finland

**Keywords:** infant and child nutrition, infant feeding, infant formula, nutritional interventions, randomized controlled trial, research methodology, type 1 diabetes

## Abstract

The international Trial to Reduce IDDM in the Genetically at Risk (TRIGR) tested the hypothesis whether extensively hydrolyzed casein‐based versus regular cow's milk‐based infant formula reduces the risk of type 1 diabetes. We describe dietary compliance in the trial in terms of study formula intake, feeding of nonrecommended foods, and serum cow's milk antibody concentration reflecting intake of cow's milk protein among 2,159 eligible newborn infants with a biological first‐degree relative affected by type 1 diabetes and with HLA‐conferred susceptibility to type 1 diabetes. The participating infants were introduced to the study formula feeding at the median age of 15 days with a median duration of study formula use of 63 days. During the intervention, 80% of the infants received study formula. Of these, 57% received study formula for at least 2 months. On average, 45.5 l of study formula were used per infant. Only 13% of the population had received a nonrecommended food by the age of 6 months. The dietary compliance was similar in the intervention and control arm. The reported cow's milk consumption by the families matched very well with measured serum casein IgA and IgG antibody concentration. To conclude, good compliance was observed in this randomized infant feeding trial. Compliance varied between the regions and those infants who were breastfed for a longer period of time had a shorter exposure to the study formula. High dietary compliance in infant feeding trial is necessary to allow accurate interpretation of study results.

## Graphical abstract

The international Trial to Reduce IDDM in the Genetically at Risk (TRIGR) tested the hypothesis whether extensively hydrolyzed casein‐based versus regular cow's milk‐based infant formula reduces the risk of type 1 diabetes. We describe good dietary compliance in the trial in terms of study formula feeding, feeding of non‐recommended foods, and serum cow's milk antibody concentration reflecting intake of cow's milk protein among 2,159 eligible newborn infants with a biological first degree relative affected by type 1 diabetes and with HLA‐conferred susceptibility to type 1 diabetes.

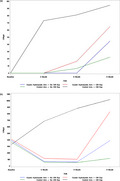

**Key messages**

- In the TRIGR study, the intervention was designed to study 2 separate groups of infants—one exposed to cow's milk protein and a nonexposed group in order to assess the effect on rates of diabetes autoimmunity and diabetes.
- Good dietary compliance was observed in this international randomized infant feeding trial whether measured by exposure to the study formulas, exposure to nonrecommended foods or by serum cow's milk protein antibodies.
- Compliance varied between the regions and those infants who received longer breastfeeding had a shorter exposure to the study formula.

## 1 INTRODUCTION

Type 1 diabetes is one of the most common chronic diseases of childhood with an increasing incidence around the world (Patterson et al., 2019). The etiology is complex and involves a genetic predisposition with suspected environmental triggers associated with autoimmune destruction of insulin‐producing beta cells (Virtanen & Knip, 2003). Certain dietary factors, along with viral infections, are among the main suspects in the etiology of type 1 diabetes (Virtanen, 2016). Of the dietary factors, early feeding of cow's milk proteins has received much research attention with inconsistent evidence from both prospective cohorts and randomized trials (Hummel et al., 2017; Knip et al., 2014, 2010; Vaarala et al., 2012; Virtanen, 2016; Writing Group for the TRIGR Study Group et al., 2018). Human studies are mostly observational and therefore confounded by issues such as breastfeeding rates and choices and selection bias. The Trial to Reduce IDDM in the Genetically at Risk (TRIGR) study reported that the cumulative incidence of diabetes‐associated autoantibodies and type 1 diabetes in children with a first‐degree relative with type 1 diabetes and a risk‐associated HLA genotype did not differ in children weaned to an extensively hydrolyzed casein formula and one containing intact cow's milk proteins (Writing Group for the TRIGR Study Group et al., 2018).

TRIGR is a randomized controlled trial launched to determine whether weaning to extensively hydrolyzed infant formula compared to one containing intact cow's milk proteins given during the first 6–8 months of life would prevent type 1 diabetes in genetically susceptible children (Knip et al., 2014; TRIGR Study Group, 2007; Åkerblom et al., 2011; Writing Group for the TRIGR Study Group et al., 2018). It is the first nutritional full‐scale randomized trial for prevention of type 1 diabetes. In this study, dairy foods were also to be avoided in the intervention period. Compliance with a dietary intervention and evaluation of variables associated with high compliance is essential factors to assess in a nutrition intervention trial. Excessive noncompliance can not only result in reduced statistical power to detect significant associations but also limit the ability to accurately interpret study results. In a pilot of the current study, very good compliance was observed: A majority of the infants (84%) were exposed to study formula for at least 2 months and the levels of cow's milk protein antibodies reflected very well cow's milk protein intake (Virtanen et al., 2011; Yang et al., 2016) examined compliance with the completion of food records in a large cohort of children in The Environmental Determinants of Diabetes in the Young (TEDDY) study. Factors associated with high compliance included older maternal age and higher maternal education while poor compliance was observed in families who lived far from the study centers and were from ethnic minority groups (Yang et al., 2016).

In the current study, we evaluate the degree of compliance to the use of study formula and to the dietary instructions on recommended and nonrecommended foods. In addition, we assess how the reported compliance is associated with the one measured by cow's milk protein antibodies. Also factors associated with the reported compliance are assessed.

## 2 METHODS

### 2.1 Recruitment and randomization

Newborn infants with a biological first‐degree relative (mother, father, or full sibling) affected by type 1 diabetes as defined by the World Health Organization were invited to participate. Recruitment strategies have been previously described in detail (TRIGR Study Group, 2007). The families were recruited when the mother was in late pregnancy (gestational age 35 weeks or more) or within 7 days of delivery. Informed consent was signed by the parents or legal guardians of the infant. Human leukocyte antigen (HLA) genotyping was performed from cord blood or from a blood sample obtained before the age of 8 days. Infants with increased HLA‐conferred susceptibility to type 1 diabetes were eligible to participate (TRIGR Study Group, 2007). Altogether 2,159 infants from 12 countries in Europe and from United States, Canada, and Australia born between May 2002 and February 2007 were included in the TRIGR study (Åkerblom et al., 2011). Of these infants, 1,095 were born to women with type 1 diabetes.

Randomization stratified by geographic area was implemented after 35 weeks of gestation or immediately after birth by the TRIGR data management unit via the TRIGR website. The TRIGR countries were divided into seven regions, Northern Europe (Estonia, Finland, and Sweden, *n* = 555), Central Europe I (Czech Republic, Hungary and Poland, *n* = 281) (transition economies), Central Europe II (Germany, Luxembourg, the Netherlands and Switzerland, *n* = 186), Southern Europe (Italy and Spain, *n* = 114), the United States (*n* = 394), Canada (*n* = 528), and Australia (*n* = 101). The early randomization allowed immediate use of study formula if breastmilk was not available. If supplemental feeding was needed prior to randomization either banked breastmilk or Nutramigen^®^ was given. Ethical approval was obtained at each study center.

Exclusion criteria included multiple gestation, an older sibling already participating in TRIGR, recognizable severe illness, gestational age <35 weeks, age of the infant more than 7 days at randomization, and no HLA sample drawn before the age of 8 days. Those infants who had received any infant formula other than the study formula or Nutramigen^®^ (Mead Johnson Nutritionals) prior to randomization, were also excluded. Finally, families having any other reasons (e.g., religious, cultural, and unwillingness) to refuse feeding the infant with cow's milk‐based products were excluded including those who planned only to breastfeed without ever using formula (TRIGR Study Group, 2007). Recruitment and retention strategies have been described in detail previously (Franciscus et al., 2014). Written informed consent was collected from all families, signed by the legal guardian of the child. The study was approved by the ethics committees of all participating centers and was conducted according to the standards of the Declaration of Helsinki and conforms to Directive 2010/63/EU.

### 2.2 Dietary intervention

Breastfeeding was encouraged. Infants were randomized to receive either an extensively hydrolyzed casein‐based infant formula (Nutramigen^®^) or a regular cow's milk‐based infant formula upon weaning from breast milk or when supplemental feeding was needed in a double‐blind fashion. Both study formulas were nutritionally complete infant formulas in powder form manufactured by one company and provided to families free of charge (Mead Johnson Nutritionals). The control formula was a mixture of commercial regular cow's milk‐based formula powder plus casein hydrolysate powder in a 4:1 ratio in order to mask the flavor and smell differences between the formulas. Formula was packaged in four different colors: two for test and two for control formula to aid in the blinding process, to provide a control for randomization during data analysis, and help to avoid accidental mis‐shipments as the families recognized their formula can color. Nutramigen^®^ or banked breast milk was used if feeding other than breast milk was needed prior to randomization or in delivery hospitals if study formula was unavailable in order to avoid exposure to intact cow's milk proteins.

The intervention lasted until the infant was 6 months old or if he/she had not received study formula at least for 2 months at that time until the age of 7–8 months. Families were counseled to avoid all other infant formulas and food products containing cow's milk or beef. Nutramigen^®^ was given to infants with suspected or proven cow's milk allergy. The use of any other infant formula (e.g., soy‐based ones) was discouraged, to maximize exposure to study formula.

During the intervention phase, four study monitors managed international coordination between European countries and Australia, and within North America. Each country had at least one coordinator and in case of several study centers, also a local coordinator in each center. Each study center had nurses and physicians with experience in pediatrics, diabetes, neonatology or obstetrics, laboratory technicians, and during the intervention period, dietitians. In each country, parents were provided lists of foods with brand names that did not contain cow's milk protein and could be used during the intervention as well as lists of nonrecommended foods containing dairy protein. Dietary advice leaflets, manual of operations, dietary interview forms, and other questionnaires were translated into 11 languages and adapted to national practices.

Information on infant feeding was acquired from the family through standardized dietary interviews. Data on the frequency of use of several allowed and nonrecommended foods were collected with a validated (Vahatalo et al., 2006) food frequency questionnaire at several time points during the first year of life. Mothers were advised and interviewed by a dietitian/study nurse by telephone when the child was 2 weeks old, 1, 2, 4, and 5 months old, and by a study dietitian during the visits at the age of 3, 6, and 9 months. When study formula feeding continued after the age of 6 months, 7 and 8 month telephone interviews were conducted by the dietitian/study nurse. Study formula exposure was defined as any reported consumption of study formula. If the child had not received the study formula for at least 60 days by 6 months of age, the intervention continued until 60 days of study formula exposure was obtained up to a maximum of 8 months of age. Noncompliance was defined as any reported exposure to nonapproved formula (anything but assigned study formula or Nutramigen^®^) or nonrecommended foods (any foods containing milk or beef proteins).

### 2.3 Measurement of cow's milk protein antibodies

IgA and IgG antibodies to cow's milk formula, beta‐lactoglobulin and alpha‐casein, were measured from serum samples obtained from cord blood and at the age of 3 and 6 months by ELISA (Savilahti et al., 1993). Cow's milk formula IgA and IgG antibodies at the age of 0, 3, 6, and 9 months are presented in the current article to show the levels in the treatment arms and by cow's milk exposure.

### 2.4 Statistical methods

The change in IgA and IgG casein antibody levels from 6 to 9 months of age was calculated by paired *t* test. Factors associated with the use of study formula were analyzed by conditional logistic regression.

## 3 RESULTS

**FIGURE 1:**
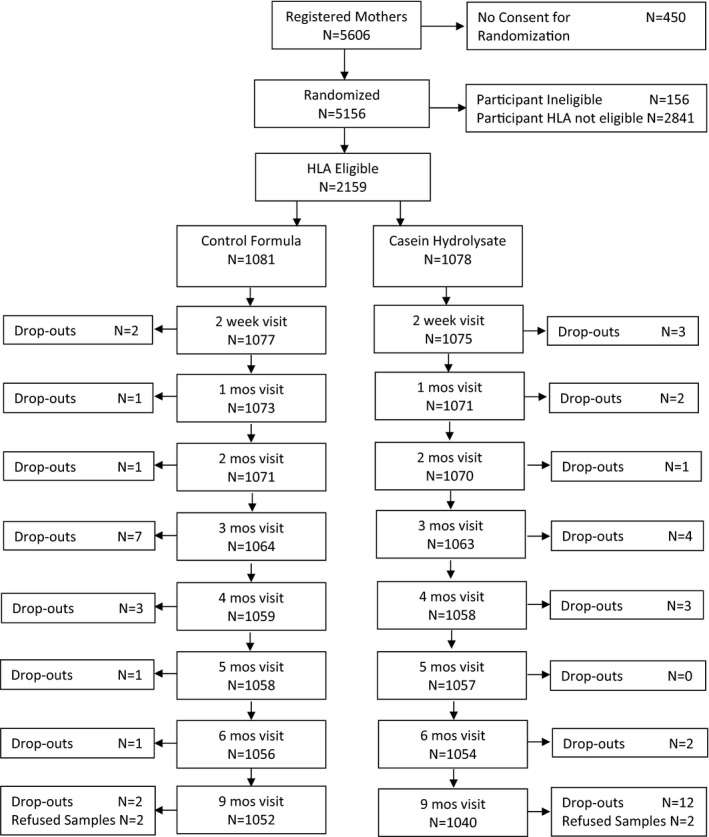
Flow chart of the trial to reduce IDDM in genetically at risk

Among the infants of the 5,156 randomized mothers, 2,159 were eligible (Figure 1). Drop‐out from the study during the intervention period was small and similar in both intervention arms (control arm, *n* = 18; casein hydrolysate, *n* = 27).

### 3.1 Rates and duration of exposure to assigned study formula

By the end of the intervention period, 80.4% of infants had received study formula and 56.7% for at least 2 months (Table 1). This did not differ between the intervention and control arms at 80.0% and 80.8%, respectively. The median age at introduction of study formula was 15 days, and the median duration of study formula use was days and did not differ between the arms. According to dietary reports on average 45.5 L of study, formula were used per child. The amount of study formula used was smaller in the casein hydrolysate than control arm (*p* =.012). At 6 months of age, fewer than 4% of infants were being exclusively breastfed.

**TABLE 1:** Study formula (SF) use by region: proportion of users, median (interquartile range, IQ) age at introduction and duration of use and mean amount of reported amount of study formula used

Region^a^	HLA eligible randomized	SF users		SF use >2 months		Age at SF introduction (days)		SF duration (days)		Amount of SF used (l)
	*N*	*N*	%	*N*	%	Median	IQ range	Median	IQ range	Mean
Australia	101	87	86.1	70	69.3	49	8–168	63	42–112	40.2
Canada	528	399	75.6	287	54.4	16	5–63	63	2–161	55.9
Central Europe I	281	236	84.0	170	60.5	31	4–168	63	21–119	40.7
Central Europe II	186	147	79.0	111	59.7	35	11–168	63	7–140	45.4
Northern Europe	555	481	86.7	332	59.8	8	3–84	63	21–126	35.4
Southern Europe	114	91	79.8	60	52.6	10	3–105	67	2–154	50.3
United States	394	295	74.9	194	49.2	16	6–60	56	2–154	49.2
Hydrolysate arm	1,081	865	80.0	595	55.0	16	4–112	63	2–126	42.3
Control arm	1,078	871	80.8	629	58.4	15	4–84	63	7–154	48.7
All	2,159	1,736	80.4	1,224	56.7	15	4–98	63	7–147	45.5

^a^ Central Europe I includes Czech Republic, Hungary, and Poland; Central Europe II includes Germany, Luxembourg, Netherlands, and Switzerland; Northern Europe includes Estonia, Finland, and Sweden; and Southern Europe includes Italy and Spain.

During the first 3 days of life, 57.8% of the infants were exclusively breastfed, 8.9% had received banked milk only, 3.5% had received study formula only, 15.1% had received Nutramigen^®^ only, 0.5% had received nonrecommended infant formula only, 13.7% had received two or more types of feeding, and 0.5% were reported not to have received any oral feeding. Of those who had received several feeding types (*n* = 290), 2.2% had received nonrecommended infant formula. As study formula feeding was not available at all the delivery hospitals, Nutramigen^®^ was considered allowed feeding straight after delivery. The early feeding did not differ between the treatment arms.

There was considerable variation in study formula use between the regions ranging from a low of 75% in North America to 86% in Australia and Northern Europe (Table 1). There was some increase in the use of study formula by time: the proportion of infants who received it for at least 2 months increased and the proportion of nonusers decreased by time (Appendix S1). The use of nonrecommended foods: cow's milk‐based infant formulas and other foods containing cow's milk or beef was similar in the treatment arms (Appendix S2). By the age of 6 months, 13% and 12% of the participating children in the intervention and control arm, respectively, had been exposed to nonrecommended foods.

### 3.2 Exposure to nonrecommended formula and foods

Exposure to Nutramigen^®^ was mostly restricted to the infants with proven or suspected study formula intolerance (data not shown). The only exception was when Nutramigen^®^ was given at the delivery hospital when breast milk or study formula was not available. Study formula intolerance was equally often suspected among children in the casein hydrolysate and control arms (data not shown). The cumulative incidence by the age of 6 months was in Central European countries I 1.4%, Southern European countries 1.9%, Central European countries II 2.2%, Northern European countries 3.8%, Canada 6.7%, Australia 7.0%, and in the United States 11.2%.

**FIGURE 2:**
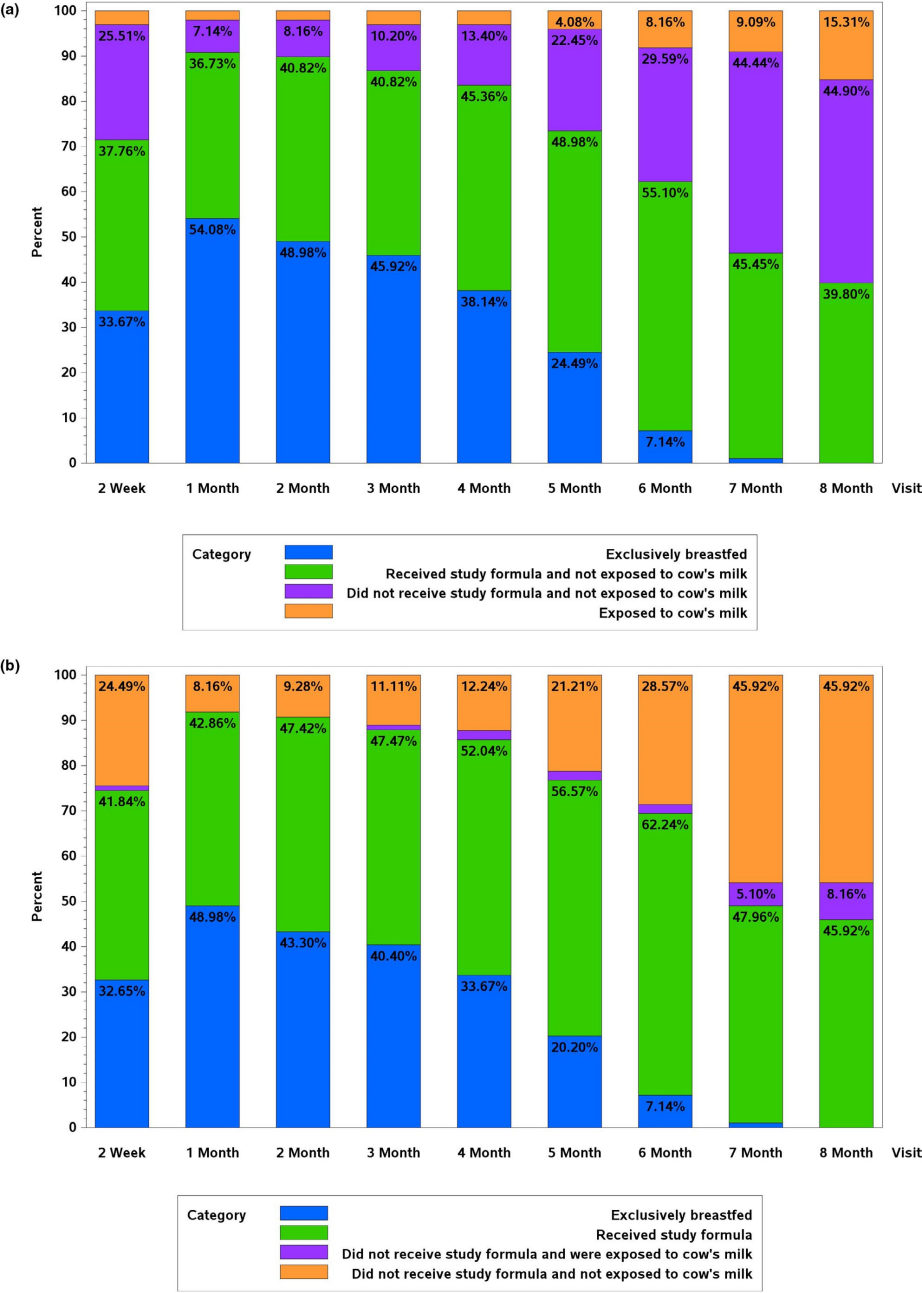
(a) Hydrolysate arm: the proportion (%) of children by type of feeding at the time of each interview. All of the categories may include breast milk. All of the children followed for at least 6 months are included (*N* = 1,054). The 7 and 8 month interview included only children who continued in the intervention up to that time: 558 and 498 children, respectively. (b) Control arm: the proportion (%) of children by type of feeding at the time of each interview. All the categories may include breast milk. All the children followed up at least for 6 months are included (*N* = 1,056). The 7 and 8 month interview included only children who continued in the intervention up to that time: 512 and 446 children, respectively

In the casein hydrolysate arm, 80.0% used the study formula during the intervention (Table 1) and most infants were reported to be unexposed to bovine protein at all the visits/calls (Figure 2a). Exposure to cow's milk proteins varied from 2.1% at 1 month of age to 8.2% at 6 months, being 15.3% at 8 months for those participants who were still in intervention at that time. Cumulatively 84.0% were not exposed to foods containing bovine protein by 6 months of age and 81.1% by the end of the intervention.

In the control arm, 80.8% were exposed to study formula during the intervention (Table 1). The proportion of infants exposed to bovine protein either in the form of study formula or nonrecommended foods varied from 42.9% during the first 2 weeks to 64.3% during the 6th month of life (Figure 2b). Cumulatively, 77.2% were exposed to bovine protein by 6 months of life and 86.7% by the end of the intervention.

**FIGURE 3:**
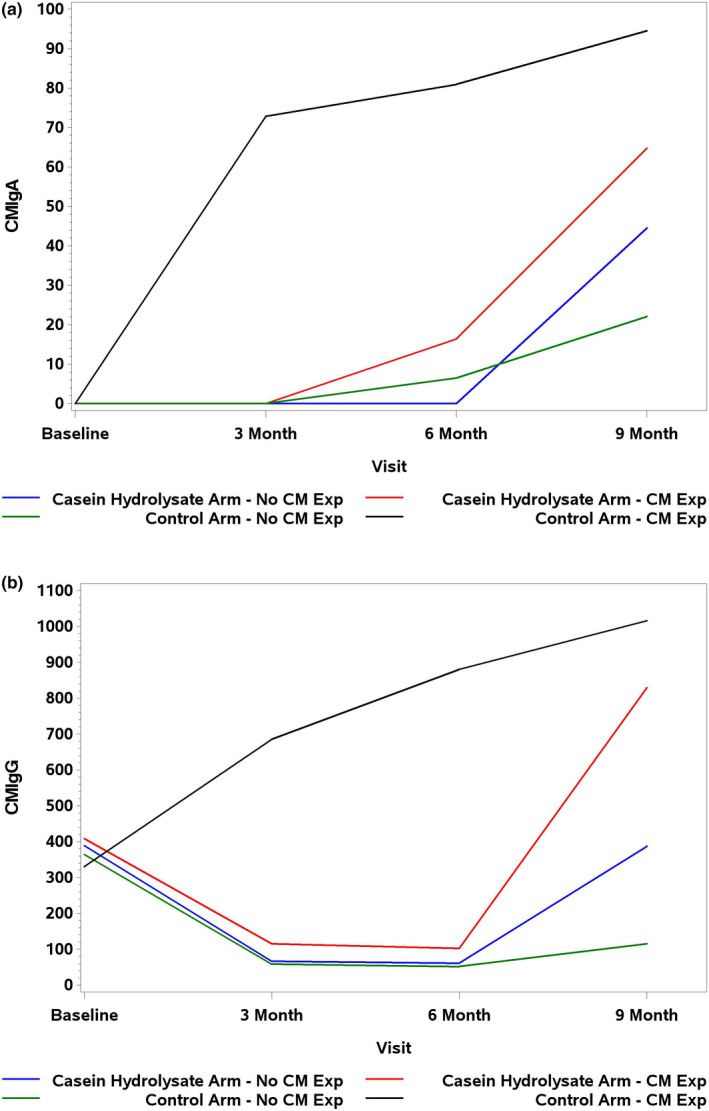
Median levels of (a) cow's milk IgA antibodies and (b) cow's milk IgG antibodies by age, intervention arm, and cow's milk (CM) exposure status

Median cow's milk IgA and IgG antibody concentrations by age, intervention arm, and cow's milk exposure are shown in Figure 3. Cow's milk IgA and IgG concentrations increased from birth among those control arm children with reported cow's milk exposure (Figure 3a,b). From 6 to 9 months, the levels of cow's milk IgA and IgG increased in all groups (*p* < .001).

Fifty‐seven percent of the infants were exposed to study formula for at least 2 months. Of the different sociodemographic and clinical factors studied only country and duration of breastfeeding were associated with at least 2 months of use of study formula in the model adjusted additionally for maternal age, education and type 1 diabetes, and sex of the child (Table 2). Being from Southern European countries, Canada and the United States compared to Northern European countries and being breastfed at least 6 months were associated with less compliance with at least 2 months study formula use (Table 2).

**TABLE 2:** Sociodemographic and clinical factors associated with compliance with at least 2 months use of study formula. Odds ratios (95% CI) for significant associations (*p* <.05) are bolded

	Odds ratio	95% CI	Adjusted odds ratio^a^	95% CI
Regions^b^ versus. Northern Europe:				
Central Europe I	1.03	0.77–1.39	0.99	0.69–1.43
Central Europe II	1.02	0.73–1.43	0.95	0.70–1.31
Southern Europe	0.75	0.50–1.12	**0.59**	**0.38–0.92**
Australia	1.52	0.97–2.40	1.60	0.99–2.59
Canada	0.81	0.63–1.03	**0.72**	**0.56–0.93**
United States	**0.65**	**0.50–0.85**	**0.60**	**0.45–0.80**
Maternal age, years	**0.97**	**0.95–0.99**	0.99	0.97–1.01
Maternal education, years	**0.93**	**0.91–0.96**	0.99	0.96–1.02
Maternal type 1 diabetes	1.18	0.99–1.40	0.92	0.76–1.11
Sex of the child: boy versus girl	1.15	0.97–1.37	1.14	0.95–1.37
Treatment: hydrolysate versus control formula	0.88	0.74–1.04	1.10	0.92–1.32
Breastfeeding: ≥6 months versus. less	**0.25**	**0.21–0.31**	**0.25**	**0.20–0.30**

^a^ The logistic regression model includes all the variables in the table.

^b^ All the regions were included at the same time in the logistic regression analysis, Northern Europe (Estonia, Finland, and Sweden) was used as reference. Central Europe I consists of Czech Republic, Hungary, and Poland), Central Europe II of Germany, Luxembourg, Netherlands, and Switzerland, and Southern Europe of Italy and Spain.

## 4 DISCUSSION

In the TRIGR study, the intervention was designed to study 2 separate groups of infants—one exposed to cow's milk protein and a nonexposed group in order to assess the effect on rates of diabetes autoimmunity and diabetes. Implementing a dietary intervention that is maintained over 6–8 months when the dietary component of interest (cow's milk protein) is very common is challenging. This report shows that such an intervention can be maintained with relatively good success. There are two main areas of interest in an intervention such as this. The first is cooperation in use of and exposure to the randomized study formula for sufficient duration. The second issue is compliance to the list of nonapproved formulae and foods. In our study, these exposures were low but increased with age of the infant. We have shown that good success can be obtained in a complex dietary intervention such as this.

Cooperation in use of the study formula, after breastfeeding, was high at 80.4% with 56.7% using study formula for at least 2 months by the end of the intervention. The intervention and control arms were similar regarding study formula use. The only exception was the slightly lower reported amount of study formula used by the intervention than control arm participants. In the current study, extensively hydrolyzed infant formula was used as the intervention tool to avoid exposure to intact cow's milk proteins. The cumulative proportion of infants not exposed to cow's milk proteins was 81% in the hydrolysate arm and the cumulative proportion of infants exposed to it was 81% in the control arm. This contrast in the exposures can be considered relatively good. Our measurement of antibodies to supplement the dietary interviews shows that the information reported by the families appeared to be reliable given that the reported cow's milk consumption was consistent with the measured casein IgA and IgG antibody concentration in serum. Breastfeeding for at least 6 months and being from Southern Europe or North America were associated with lower rates of exposure to study formula for at least 2 months.

In the hydrolysate study formula arm, unintended exposure to cow's milk proteins was very low: varying from 2% at 1 month of age to 8% at 6 months, and being 15% at 8 months for those participants who were still in the intervention. Exposure to nonapproved formula and foods was similarly low in the control arm (0% at 1 month, 2% at 6 months, and 8% at 8 months). The dietary interview forms used were validated (Vahatalo et al., 2006) and translated into all the languages of the study countries making it likely that data collected regarding noncompliance is complete.

The drop‐out rates by the age of 2 years in infant feeding trials using hydrolyzed and long‐chain polyunsaturated fatty acid‐containing formulas have varied between 16% and 30% (Kalliomaki et al., 2001; Makrides et al., 1999; Mallet & Henocq, 1992; Marini et al., 1996; Zeiger et al., 1989). In infant feeding trials, relatively good dietary compliance has been observed. In a Danish allergy avoidance study, 6.2% of the 550 infants received another formula than originally randomized to (Halken et al., 2000). In a Swiss allergy prevention study, dietary noncompliance rate was 8.4% including the use of other infant formulas and weaning foods than recommended (Exl et al., 2000). In a German study on allergy prevention, of the 2,252 infants randomized to one of the four formulas, 58% received the study formula and 11% made deviations in the advised diet (von Berg et al., 2003). In another German study, Schoetzau et al. (2002) examined maternal compliance with nutritional recommendations in an allergy prevention trial and reported factors that influenced compliance. The intervention was similar to the TRIGR trial in that families were advised to use the study formula if breastfeeding was not possible and to avoid the introduction of solid foods before 4 months of age. The authors reported a drop‐out rate of 13.5% during the first year of life and a high compliance (defined as completion of all infant diaries and adherence to all nutritional recommendations) rate of 83.4% between weeks 1 and 16. In comparison, the TRIGR study observed a 3% drop‐out rate at 1 year and had an 80.4% compliance rate at the completion of the intervention.

Compared to the previous TRIGR pilot study, a smaller proportion of the infants received study formula, for example, the proportion of infants with at least 2 months study formula exposure was 57% in the current study compared to 84% in the pilot study (Virtanen et al., 2011). In the current study, the introduction of study formula happened earlier (median 2 weeks of age compared to 2 months in the pilot) and the duration of observed study formula use was shorter (median 2 months versus 4 months, respectively). The difference in the length of the study formula use between the studies was partly explained by the fact that longer than recommended use of study formula was rather common in the pilot study, 33% of the infants receiving study formula longer than recommended (Virtanen et al., 2011). The reported proportion of families who deviated from the recommended diet by giving cow's milk‐based infant formulas other than the study formula and/or other nonrecommended foods containing cow's milk or beef were similarly moderate in the current and pilot studies (18% in both).

There are a number of factors in this study that may have led to this degree of success in maintaining separation between the study arms. The study had a rigorous double‐blind randomized trial design. Blinding was facilitated by the addition of 20% of casein hydrolysate also to the control formula, so the study formulas could not be differentiated by taste or smell. The families were contacted frequently during the intervention and the drop‐out up was small. The availability of national and international coordinators (some of them nutritionists) and dietitians, helped to maintain regular training of the personnel, coordination of the training between the regions and rapid responses to queries from the sites (Franciscus et al., 2014). A manual of operations contained detailed description of study procedures including recruitment, training, advising of the families, advice material, forms, and study conduct. We have shown earlier that the intervention did not adversely affect breastfeeding habits (Sorkio et al., 2010). It could also be that the families who knew the child's HLA risk invested in the study demands.

This study has several limitations. Although our retention rates exceeded planning parameters and the amount of missing information was very low, there is the possibility that participation in a nutrition intervention trial influenced families’ feeding choices. Compliance rates may also have been influenced by confounding factors that were not observed. Given the inclusion and exclusion criteria for the TRIGR study, the results may not be generalizable to children without increased HLA‐conferred susceptibility for T1D.

The cumulative proportion of infants with suspected cow's milk intolerance in the current study (approximately 5%–6% by the age of 8 months) was clearly above expected levels of 2%–3% of infants based on strict diagnostic criteria of cow's milk allergy in developed countries (Host & Halken, 2014). Further, unexpectedly, it did not differ between the intervention arms. The method of measurement of intolerance used in the TRIGR centers was mainly open challenge test either at clinic or at home, but some families were unwilling to participate in the challenge.

In the TRIGR pilot study, the use of extensively hydrolyzed infant formula decreased the incidence of islet cell autoantibodies, whereas in the current study no effect on islet autoimmunity was seen (Knip et al., 2010, 2014). The earlier and smaller exposure to study formulas in the current than in the pilot study needs to be considered as one possible explanation when interpreting the inconsistent findings of the two studies.

To conclude, good dietary compliance was observed in an international randomized infant feeding trial whether measured by exposure to the study formulas, exposure to nonrecommended foods or by cow's milk protein antibodies. Compliance varied between the regions and those infants who received longer breastfeeding had a shorter exposure to the study formula.

## CONFLICTS OF INTEREST

The authors had no conflicts of interest.

## AUTHOR CONTRIBUTIONS

Suvi M. Virtanen: Conceptualization (lead); Data curation (supporting); Funding acquisition (supporting); Investigation (lead); Methodology (lead); Project administration (supporting); Supervision (equal); Writing‐original draft (lead). David Cuthbertson: Data curation (lead); Formal analysis (lead); Investigation (equal); Methodology (equal); Software (lead); Writing‐review & editing (equal). Anita M. Nucci: Conceptualization (equal); Data curation (equal); Investigation (equal); Methodology (equal); Project administration (supporting); Writing‐review & editing (equal). Mila Hyytinen: Data curation (equal); Investigation (supporting); Writing‐review & editing (supporting). Anne Ormisson: Data curation (supporting); Investigation (supporting); Writing‐review & editing (supporting). Marja Salonen: Data curation (supporting); Investigation (supporting); Project administration (supporting); Writing‐review & editing (supporting). Tania Turrini: Investigation (supporting); Writing‐review & editing (supporting). Elizabeth A. Cummings: Investigation (supporting); Writing‐review & editing (supporting). Brenda Bradley: Data curation (supporting); Investigation (supporting); Writing‐review & editing (supporting). Marilyn Tanner‐Blasiar: Investigation (supporting); Writing‐review & editing (supporting). Dorothy Becker: Conceptualization (supporting); Data curation (supporting); Funding acquisition (equal); Investigation (supporting); Project administration (supporting); Writing‐review & editing (equal). Hans K. Åkerblom: Conceptualization (supporting); Funding acquisition (equal); Investigation (equal); Writing‐review & editing (supporting). Erkki Savilahti: Conceptualization (supporting); Data curation (supporting); Investigation (equal); Writing‐review & editing (supporting). Jeffrey P. Krisher: Conceptualization (supporting); Data curation (equal); Funding acquisition (equal); Investigation (supporting); Project administration (equal); Supervision (supporting); Writing‐review & editing (supporting). Mikael Knip: Conceptualization (equal); Data curation (supporting); Funding acquisition (lead); Investigation (supporting); Project administration (equal); Supervision (supporting); Writing‐review & editing (equal).

## Supporting information

Appendix S1

Appendix S2

Supplementary Material
