# Supplementary figures and images for: Dietary compliance in a randomized double‐blind infant feeding trial during infancy aiming at prevention of type 1 diabetes
Source: Food Sci Nutr. 2021 Jun 23;9(8):4221–31. doi: 10.1002/fsn3.2389 (PMC8358383; doi:10.1002/fsn3.2389)

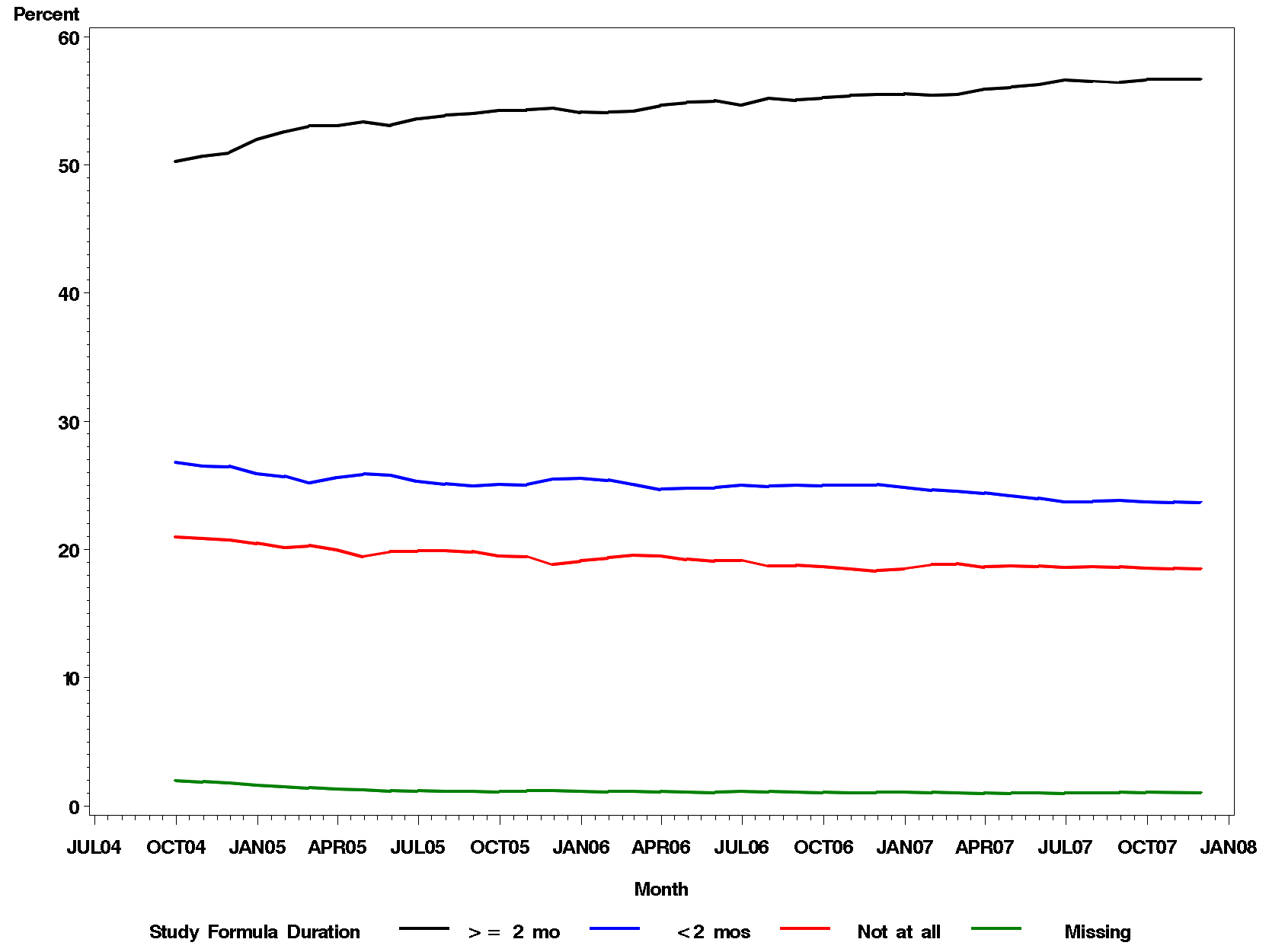

Supplement: Supplementary file 1 — Appendix S1 [file FSN3-9-4221-s001.tif]

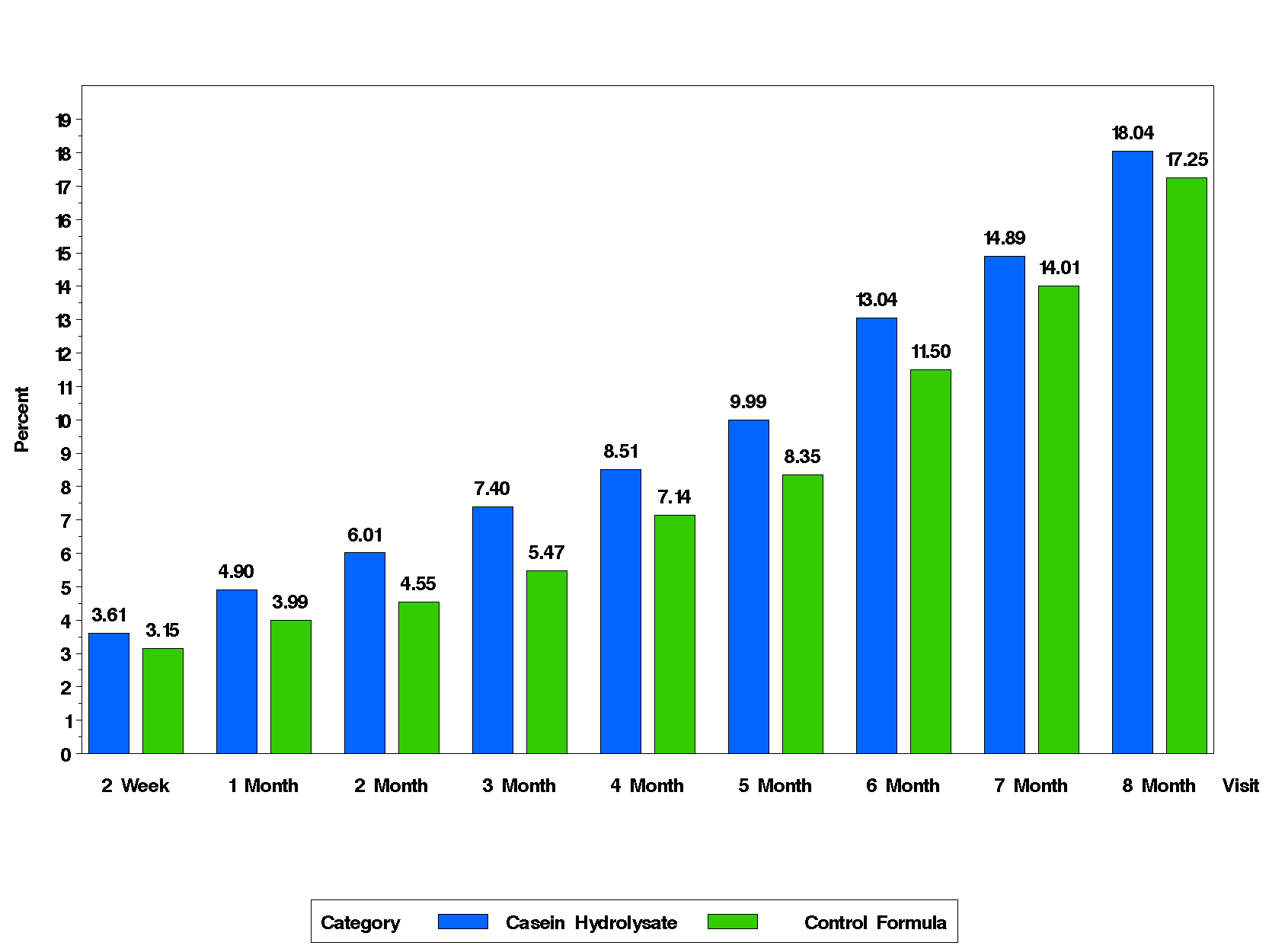

Supplement: Supplementary file 2 — Appendix S2 [file FSN3-9-4221-s003.tif]
