# Supplementary material for: Dietary compliance in a randomized double‐blind infant feeding trial during infancy aiming at prevention of type 1 diabetes
Source: Food Sci Nutr. 2021 Jun 23;9(8):4221–31. doi: 10.1002/fsn3.2389 (PMC8358383; doi:10.1002/fsn3.2389)
Supplement: Supplementary file 3 — Supplementary Material [file FSN3-9-4221-s002.docx]

| **Data Safety Monitoring Board** |  | Mandrup-Poulsen | Thomas | Chair |
| --- | --- | --- | --- | --- |
|  |  | Arjas | Elias | Member |
|  |  | Läärä | Esa | Member |
|  |  | Lernmark | Åke | Member |
|  |  | Schmidt | Barbara | Member |
|  |  | Krischer | Jeffrey P. | Observer |
|  |  |  |  |  |
| **International Coordinating Center (ICC), Helsinki, Finland** |  | Åkerblom | Hans K. † * | PI of the Study until 30.6.08, Deputy PI from 1.7.2008 |
|  |  | Hyytinen | Mila | European Study Monitor |
|  |  | Knip | Mikael | Deputy PI until 30.6.2008, PI of the Study from 1.7.2008, National Investigator |
|  |  | Koski | Katriina | European Study Monitor |
|  |  | Koski | Matti | IT Specialist |
|  |  | Pajakkala | Eeva | European Study Monitor |
|  |  | Salonen | Marja | Study Coordinator |
|  |  |  |  |  |
| **Data Management Unit (DMU), Tampa, FL, USA** |  | Cuthbertson | David | Biostatistician |
|  |  | Krischer | Jeffrey P. | PI of the DMU |
|  |  | Shanker | Linda | Coordinator |
|  |  |  |  |  |

| **Canadian Coordinating Center, London and Ottawa, ON** |  | Bradley | Brenda | National Coordinator |
| --- | --- | --- | --- | --- |
|  |  | Dosch | Hans-Michael | Co-Investigator for Canada |
|  |  | Dupré | John | Co-PI for North America and  National Investigator until 08/12/2015,  Executive Committee |
|  |  | Fraser | William | Co-Investigator for Canada  Executive Committee |
|  |  | Lawson | Margaret | Co-Investigator for Canada  Executive Committee |
|  |  | Mahon | Jeffrey L. | Co-PI for North America and  National Investigator after 08/12/2015,  Executive Committee |
|  |  | Sermer | Mathew | Co-Investigator for Canada,  Executive Committee |
|  |  | Taback | Shayne P. | Co-Investigator for Canada,  Executive Committee |
|  |  |  |  |  |
| **USA Coordinating Center, Pittsburgh, PA and Seattle, WA** |  | Becker | Dorothy | Co-PI for North America,  National Investigator, Executive Committee |
|  |  | Franciscus | Margaret | National Coordinator |
|  |  | Nucci | Anita | National Coordinator, Nutrition Coordinator of North America |
|  |  | Palmer | Jerry | Executive Committee |
|  |  |  |  |  |
| **Nutritional Epidemiology Unit, Helsinki, Finland** |  | Virtanen | Suvi M. | Head of Nutritional Epidemiology Unit |
|  |  |  |  |  |
|  |  |  |  |  |
| **Australia** | **AUS01 - Westmead - Children’s Hospital** | Catteau | Jacki | National Coordinator |
|  |  | Howard | Neville | National Investigator |
|  |  |  |  |  |
|  | **AUS02 - Newcastle - John Hunter Children’s Hospital** | Crock | Patricia | Local Investigator |
|  |  |  |  |  |
|  | **AUS03 - Sydney - Sydney Children’s Hospital** | Craig | Maria | Local Investigator |
|  |  |  |  |  |
| **Canada** | **CAN01 - London - St. Joseph's Health Care Centre** | Clarson  Bere | Cheril L.  Lynda | Local Investigator  Coordinator |
|  |  |  |  |  |
|  | **CAN02 - Vancouver - Children's and Women's Health Centre of British Columbia** | Thompson | David | Local Investigator |
|  |  | Metzger | Daniel | Local Investigator |
|  |  | Marshall  Kwan | Colleen  Jennifer | Coordinator (In Transition)  Coordinator (In Transition) |
|  |  |  |  |  |
|  | **CAN03 - Calgary - Alberta Children's Hospital** | Stephure | David K. | Local Investigator |
|  |  | Pacaud  Schwarz | Daniele  Wendy | Co-Investigator  Coordinator |
|  |  |  |  |  |
|  | **CAN04 - Edmonton - Walter MacKenzie Health Sciences** | Girgis  Thompson | Rose  Marilyn | Local Investigator  Coordinator |
|  |  |  |  |  |
|  | **CAN05 - Winnipeg - Health Sciences Centre** | Taback  Catte | Shayne P  Daniel | Local Investigator  Coordinator |
|  |  |  |  |  |
|  | **CAN06 - Ottawa - Children's Hospital of Eastern Ontario and The Ottawa Hospital** | Lawson  Bradley | Margaret L  Brenda | Local Investigator  Coordinator |
|  |  |  |  |  |
|  | **CAN07 - Toronto Mount Sinai Hospital/Hospital for Sick Children** | Daneman | Denis | Local Investigator |
|  |  | Sermer  Martin | Mathew  Mary-Jean | Co-Investigator  Coordinator |
|  |  |  |  |  |
|  | **CAN08 - Quebec - CHUQ** | Morin | Valérie | Local Investigator |
|  |  | Frenette  Ferland | Line  Suzanne | Co-Investigator  Coordinator |
|  |  |  |  |  |
|  | **CAN09 - Saint John – Regional Hospital** | Sanderson  Heath | Susan  Kathy | Local Investigator  Coordinator |
|  |  |  |  |  |
|  | **CAN10 - Montreal - L' Hôpital Sainte-Justine** | Huot | Céline | Local Investigator |
|  |  | Gonthier  Thibeault | Monique  Maryse | Co-Investigator  Coordinator |
|  |  |  |  |  |
|  | **CAN11 - Montreal Children's Hospital** | Legault  Laforte | Laurent  Diane | Local Investigator  Coordinator |
|  |  |  |  |  |
|  | **CAN12 - Halifax - IWK Health Centre/Dalhousie** | Cummings  Scott | Elizabeth A  Karen | Local Investigator  Coordinator |
|  |  |  |  |  |
|  | **CAN13 - St. John's - Janeway Child Health Center** | Bridger  Crummell | Tracey  Cheryl | Local Investigator  Coordinator |
|  |  |  |  |  |
|  |  |  |  |  |
|  | **CAN14 - Kingston - Kingston General Hospital/ Queen's University** | Houlden  Breen | Robyn  Adriana | Local Investigator  Coordinator |
|  |  |  |  |  |
|  | **CAN15 - Regina - Regina Qu'Appelle** | Carson  Kelly | George  Sheila | Local Investigator  Coordinator |
|  |  |  |  |  |
|  | **CAN16 - Saskatoon - Royal University Hospital** | Sankaran  Penner | Koravangattu  Marie | Local Investigator  Coordinator |
|  |  |  |  |  |
|  | **CAN17 - Peterborough - Peterborough Regional Health Centre** | White  King | Richard A  Nancy | Local Investigator  Coordinator |
|  |  |  |  |  |
|  | **CAN18 - Victoria - Vancouver Island Health Research Centre** | Popkin  Robson | James  Laurie | Local Investigator  Coordinator |
|  |  |  |  |  |
| **Czech Republic** | **CZE01 - Prague - Faculty Hospital Kralovske Vinohrady** | Al Taji | Eva | National Investigator/Coordinator |
|  |  | Mendlova | Pavla | National Coordinator |
|  |  | Romanova | Martina | Co-Investigator |
|  |  | Vavrinec | Jan † * | National Investigator |
|  |  | Vosahlo | Jan | Co-Investigator |
|  |  |  |  |  |
|  | **CZE02 - Brno - Hospital Milosrdnych Bratri** | Brazdova | Ludmila | Local Investigator |
|  |  |  |  |  |
|  | **CZE03- Olomouc - Faculty Hospital Olomouc** | Venhacova | Jitrenka | Local Investigator |
|  |  | Venhacova | Petra | Co-Investigator |
|  |  |  |  |  |
|  | **CZE04 - Usti nad Labem - Hospital of Masryk** | Cipra | Adam | Local Investigator |
|  |  |  |  |  |
|  | **CZE05 - Ceske Budejovice - Hospital Ceske Budejovice** | Tomsikova | Zdenka | Local Investigator |
|  |  |  |  |  |
|  | **CZE06 - Plzen - Faculty Hospital Plzen** | Paterová | Petra | Local Investigator |
|  |  |  |  |  |
|  | **CZE07 - Zlin - Hospital of Bata** | Gogelova | Pavla | Local Investigator |
|  |  |  |  |  |
| **Estonia** | **EST01 - Tallinn - Tallinn Children’s Hospital** | Einberg | Ülle | Co-Investigator |
|  |  | Riikjärv | Mall-Anne | Local Investigator |
|  |  |  |  |  |
|  | **EST02 - Tartu - Tartu University Children’s Hospital** | Ormisson | Anne | National Investigator |
|  |  | Tillmann | Vallo | Co-Investigator |
|  |  |  |  |  |
| **Finland** | **FIN01 - Helsinki – Children’s Hospital, University of Helsinki** | Johansson | Susanne | National Coordinator |
|  |  | Kleemola | Päivi | National Coordinator |
|  |  | Parkkola | Anna | Local Investigator |
|  |  |  |  |  |
|  | **FIN02 - Helsinki - Department of Obstetrics and Gynecology, University of Helsinki** | Järvenpää | Anna-Liisa | Local Investigator |
|  |  |  |  |  |
|  | **FIN03 - Espoo - Jorvi Hospital** | Hämäläinen | Anu-Maaria | Local Investigator |
|  |  | Kiiveri | Sanne | Local Investigator |
|  | **FIN04 - Kotka - Kymenlaakso Central Hospital** | Salonen | Maria | Local Investigator |
|  |  | Tenhola | Sirpa | Local Investigator |
|  |  |  |  |  |
|  |  |  |  |  |
|  | **FIN05 - Lahti - Paijat-Hame Central Hospital** |  |  |  |
|  |  | Salonen | Pia | Local Investigator |
|  |  |  |  |  |
|  | **FIN06 - Tampere - Department of Pediatrics, Tampere University Hospital** | Jason | Eeva | Local Investigator |
|  |  | Selvenius | Jenni | Local Investigator |
|  |  | Siljander | Heli | Co-Investigator |
|  |  |  |  |  |
|  | **FIN07 - Pori - Satakunta Central Hospital** |  |  |  |
|  |  | Ylitalo | Samuli | Local Investigator |
|  |  |  |  |  |
|  | **FIN08 - Jyväskylä - Central Finland Central Hospital** |  |  |  |
|  |  | Paajanen | Ilkka | Local Investigator |
|  |  |  |  |  |
|  | **FIN09 - Seinäjoki - South Ostrobotnia Central Hospital** | Talvitie | Timo | Local Investigator |
|  |  |  |  |  |
|  | **FIN10 - Hyvinkää - Hyvinkää Hospital** | Lindström | Kaija | Local Investigator |
|  |  |  |  |  |
|  | **FIN11 - Kuopio - Department of Pediatrics, Kuopio University Hospital** | Huopio | Hanna | Local investigator |
|  |  | Pesola | Jouni | Co-Investigator |
|  |  |  |  |  |
|  | **FIN12 - Oulu - Department of Pediatrics, Oulu University Hospital** | Veijola | Riitta | Local Investigator |
|  |  | Tapanainen | Päivi | Co-Investigator |
|  |  |  |  |  |
|  |  |  |  |  |
|  | **FIN13 - Hämeenlinna - Kanta-Hame Central Hospital** | Alar | Abram | Local Investigator |
|  |  |  |  |  |
|  |  |  |  |  |
|  | **FIN14 - Vaasa - Vaasa Central Hospital** | Popov | Erik | Local Investigator |
|  |  |  |  |  |
|  |  |  |  |  |
|  | **FIN15 - Lappeenranta - South Carelian Central Hospital** | Virransalo | Ritva | Local Investigator |
|  |  |  |  |  |
|  | **FIN16 - Mikkeli - Mikkeli Central Hospital** | Nykänen | Päivi | Local Investigator |
|  |  |  |  |  |
| **Germany** | **GER01 - Hannover - Kinder- und Jugendkrankenhaus – Auf der Bult** | Aschemeier | Bärbel | National Coordinator |
|  |  | Danne | Thomas | National Investigator |
|  |  | Kordonouri | Olga | Co-Investigator |
|  |  |  |  |  |
| **Hungary** | **HUN01 - Budapest - Semmelweis Medical University** | Krikovszky | Dóra | Co-Investigator |
|  |  | Madácsy | László | National Investigator |
|  |  |  |  |  |
| **Italy** | **ITA01 - Rome - University Campus Bio-Medico of Rome** | Khazrai | Yeganeh Manon | Local Coordinator |
|  |  | Maddaloni | Ernesto | Local Coordinator |
|  |  | Pozzilli | Paolo | National Investigator |
|  |  |  |  |  |
|  | **SAR01 - Cagliari - St. Michele Hospital** | Mannu | Carla | Local Coordinator |
|  |  | Songini | Marco | National Investigator |
|  |  |  |  |  |
| **Luxembourg** | **LUX01 - Luxembourg - Centre Hospitalier de Luxembourg** | de Beaufort | Carine | National Investigator |
|  |  | Schierloh | Ulrike | Co-Investigator |
|  |  |  |  |  |
| **The Netherlands** | **NET01 - Rotterdam - Sophia Children’s Hospital** | Bruining | Jan † * | National Investigator |
|  |  | Bisschoff | Margriet | National Coordinator |
|  |  |  |  |  |
|  |  |  |  |  |
| **Poland** | **POL01 - Wroclaw - Medical University of Wroclaw** | Basiak | Aleksander | Co-Investigator |
|  |  | Wasikowa | Renata | National Investigator |
|  |  |  |  |  |
|  | **POL02 - Krakow - Polish-American Children’s Hospital** | Ciechanowska | Marta | Local Investigator |
|  |  |  |  |  |
|  |  |  |  |  |
|  | **POL03 - Katowice - Medical University of Silesia** | Deja | Grazyna | Co-Investigator |
|  |  | Jarosz-Chobot | Przemyslawa | Local Investigator |
|  |  |  |  |  |
|  | **POL04 - Lodz - Medical University of Lodz** | Szadkowska | Agnieszka | Co-Investigator |
|  |  |  |  |  |
|  | **POL05 - Lodz - Polish Mother’s Memorial Hospital (I.C.Z.M.P)** | Cypryk | Katarzyna | Local Investigator |
|  |  | Zawodniak-Szalapska | Malgorzata | Co-Investigator |
|  |  |  |  |  |
| **Spain** | **SPA01 - Cruces University Hospital-UPV/EHU-CIBERDEM/CIBERER, Barakaldo, Spain** | Castano | Luis | National Investigator |
|  |  | Chueca | Maria | Co-Investigator |
|  |  | Gonzalez Frutos | Teba | Local Coordinator |
|  |  |  |  |  |
|  |  |  |  |  |
|  |  |  |  |  |
|  | **SPA02 - Madrid - Hospital Clinico San Carlos** | Serrano-Ríos | Manuel | National Investigator |
|  |  | Martínez-Larrad | María Teresa | Local Coordinator |
|  |  | Hawkins | Federico Gustavo | Co-Investigator |
|  |  |  |  |  |
|  | **SPA03 - Madrid - Hospital Gregorio Marañon** | Rodriguez Arnau | Dolores | Co-Investigator |
|  |  |  |  |  |
|  |  |  |  |  |
| **Sweden** | **SWE01 - Linköping -**  **University of Linköping** | Ludvigsson | Johnny | National Investigator |
|  |  | Smolinska Konefal | Malgorzata | National Coordinator |
|  |  |  |  |  |
|  | **SWE02 - Uddevalla - Uddevalla Hospital** | Hanas | Ragnar | Local Investigator |
|  |  |  |  |  |
|  | **SWE03 - Göteborg - GothenburgThe Queen Silvia Children’s Hospital** | Lindblad | Bengt | Local Investigator |
|  |  |  |  |  |
|  | **SWE05 - Halmstad - Halmstad Hospital** | Nilsson | Nils-Östen | Local Investigator |
|  |  |  |  |  |
|  | **SWE06 - Trollhättan - Trollhättan Hospital** | Fors | Hans | Local Investigator |
|  |  |  |  |  |
|  | **SWE07 - Norrköping - Vrinnevi Hospital** | Nordwall | Maria | Local Investigator |
|  |  |  |  |  |
|  | **SWE08 - Borås - Borås Hospital** | Lindh | Agne | Local Investigator |
|  |  |  |  |  |
|  | **SWE09 - Karskrona - Karlskrona Hospital** | Edenwall | Hans | Local Investigator |
|  |  |  |  |  |
|  | **SWE10 - Örebro - University Hospital** | Åman | Jan | Local Investigator |
|  |  |  |  |  |
|  | **SWE11 - Jönköping - Ryhovs Hospital** | Johansson | Calle | Local Investigator |
|  |  |  |  |  |
| **Switzerland** | **SWT01 - Zürich - University Children’s Hospital** | Gadient | Margrit | Local Coordinator |
|  |  | Konrad | Daniel | National Investigator |
|  |  | Schoenle | Eugen | National Investigator |
|  |  |  |  |  |
| **USA** | **USA01 - Pittsburgh - Children's Hospital of Pittsburgh** | Becker | Dorothy | USA National Investigator / Pittsburgh Local Investigator |
|  |  | Daftary | Ashi | Co-Investigator |
|  |  | Klein | Mary Beth | Pittsburgh  Coordinator |
|  |  | Gilmour | Carol | Co-Investigator |
|  |  |  |  |  |
|  | **USA02 - Seattle - VA Puget Sound Health Care System and University of Washington** | Palmer | Jerry | Local Investigator |
|  |  | Malone | Patty | Coordinator |
|  |  |  |  |  |
|  |  |  |  |  |
|  | **USA03 - St. Louis - Washington University** | Tanner-Blasiar | Marilyn | Coordinator |
|  |  | White | Neil | Local Investigator |
|  |  |  |  |  |
|  | **USA04 - Los Angeles - Mattel Children's Hospital of UCLA** | Devaskar | Uday | Local Investigator |
|  |  | Horowitz | Heather | Coordinator/dietitian |
|  |  | Rogers | Lisa | Coordinator/dietitian |
|  |  |  |  |  |
|  | **USA05 - Ponce - Ponce School of Medicine**  **USA06 - New York - Naomie Berrie Diabetes Center** | Colon | Roxana | Coordinator |
|  |  | Frazer | Teresa | Co-Investigator |
|  |  | Torres | Jose | Local Investigator |
|  |  |  |  |  |
|  |  | Goland | Robin | Local Investigator |
|  |  | Greenberg | Ellen | Coordinator |
|  |  | Schachner | Holly | Co-Investigator |
|  |  | Softness | Barney | Co-Investigator |
|  |  |  |  |  |
| **Laboratories** | **HLA-typing Laboratory - Turku - Finland** | Ilonen | Jorma | Head of HLA-typing Laboratory |
|  |  |  |  |  |
|  | **HLA-typing Laboratory - Pittsburgh - PA - USA** | Trucco | Massimo | Head of HLA-typing Laboratory |
|  |  | Nichol | Lynn | Chief Technician |
|  |  |  |  |  |
|  | **Cow's Milk Antibody Laboratory - Helsinki - Finland** | Savilahti | Erkki | Head of Cow's Milk Antibody Laboratory |
|  |  |  |  |  |
|  | **Autoantibody Laboratory - Helsinki – Finland** | Härkönen | Taina | Co-Investigator |
|  |  | Knip | Mikael | Head of Antibody Laboratory |
|  |  |  |  |  |
|  | **T-Cell Laboratory - Helsinki – Finland** | Vaarala | Outi | Head of T-cell Laboratory |
|  |  | Luopajärvi | Kristiina | Co-Investigator |
|  |  |  |  |  |
|  | **T-Cell Laboratory - Toronto - ON - Canada** | Dosch | Hans-Michael | Head of T-Cell Laboratory |
|  |  |  |  |  |
